# Outcomes, Sequelae, and Ventilatory Strategies in Long COVID Patients with Severe ARDS: A Retrospective Cohort Study

**DOI:** 10.3390/jcm14207223

**Published:** 2025-10-13

**Authors:** Diana-Alexandra Mîțu, Florina Buleu, Daian-Ionel Popa, Cosmin Trebuian, Dumitru Sutoi, Adina Coman, Daniel Florin Lighezan, Tiberiu Buleu, Natheer Sliman, Oana Raluca Radbea, Ovidiu Alexandru Mederle

**Affiliations:** 1 Doctoral School, Faculty of General Medicine, “Victor Babes” University of Medicine and Pharmacy, 300041 Timisoara, Romania; diana-alexandra.mitu@umft.ro (D.-A.M.);; 2 Department of Cardiology, “Victor Babes” University of Medicine and Pharmacy, 300041 Timisoara, Romania; 3 Emergency Municipal Clinical Hospital, 300254 Timisoara, Romania; 4 Research Center for Medical Communication, “Victor Babes” University of Medicine and Pharmacy, 300041 Timisoara, Romania; 5 Department of Surgery, Emergency Discipline, “Victor Babes” University of Medicine and Pharmacy, 300041 Timisoara, Romaniadumitru.sutoi@umft.ro (D.S.); 6 Faculty of Nursing, “Victor Babes” University of Medicine and Pharmacy, 300041 Timisoara, Romania; 7 County Emergency Clinical Hospital, 550245 Sibiu, Romania; 8 Faculty of General Medicine, “Victor Babes” University of Medicine and Pharmacy, 300041 Timisoara, Romania

**Keywords:** COVID-19, acute respiratory distress syndrome, ventilation treatment, high-flow nasal cannula, continuous positive airway pressure, non-invasive ventilation, endotracheal intubation

## Abstract

**Background and Aims:** Severe acute respiratory distress syndrome (ARDS) in patients with long COVID remains associated with extremely high mortality and significant long-term sequelae. Non-invasive ventilatory strategies such as continuous positive airway pressure (CPAP) and high-flow nasal cannula (HFNC) are widely used before endotracheal intubation (ETI). Still, their comparative effectiveness in this population is not well established. Understanding survival outcomes and sequelae can help refine treatment strategies for this high-risk group. This study aimed to evaluate outcomes, sequelae, and treatment strategies in long COVID patients with severe ARDS, focusing on non-invasive ventilatory support before ETI. **Materials and Methods:** A retrospective cohort analysis was performed using a study comparing severe ARDS patients with and without COVID-19. The inclusion criterion was a Horovitz quotient (PaO_2_/FiO_2_) < 50 mmHg. **Results:** The study included a total of 59 patients diagnosed with long COVID-19 ARDS, with a mortality rate of 85%. A significant proportion of the patient population was male, accounting for 75%. The highest survival rate was observed among patients who initially received CPAP support, with a survival rate of 23.08%, in contrast to those treated solely with HFNC or those who alternated between HFNC and CPAP. Among patients who required endotracheal intubation and subsequent mechanical ventilation, survival rates were 40% for those who had previously received CPAP, 10% for those treated with alternating HFNC and CPAP, and 0% for those managed exclusively with HFNC before ETI. Survivors often exhibited sequelae, such as impaired pulmonary function, persistent dyspnea, and diminished physical performance. **Conclusions:** Patients with long COVID who develop severe ARDS (PaO_2_/FiO_2_ < 50 mmHg) face exceptionally high ICU mortality, with outcomes determined mainly by age, comorbidities, and profound hypoxemia. Although CPAP showed a trend toward improved survival, the data do not establish superiority and should be regarded as hypothesis-generating. Rather, they highlight the complexity of managing this underrepresented subgroup and underscore the need for larger, multicenter studies with broader inclusion criteria to confirm or refute these preliminary observations.

## 1. Introduction

Coronavirus disease 2019 (COVID-19), caused by severe acute respiratory syndrome coronavirus 2 (SARS-CoV-2), continues to present significant challenges in critical care medicine. One of the most severe complications associated with this disease is acute respiratory distress syndrome (ARDS), which occurs in a considerable number of patients suffering from critical COVID-19 and is linked to alarmingly high mortality rates [1,2].

An important subset of patients affected by COVID-19 are those with long COVID, also referred to as post-COVID-19 condition. According to the World Health Organization, long COVID is defined as a set of symptoms occurring within three months of acute SARS-CoV-2 infection and persisting for at least two months, without an alternative explanation [3]. Clinical manifestations are highly heterogeneous and commonly include fatigue, dyspnea, and cognitive impairment, with more than 200 symptoms described to date. Integrating this definition with the appropriate references would improve the reader’s understanding of the clinical background and the study population [3,4,5].

Patients with long COVID often exhibit reduced pulmonary reserve and consequently face an increased risk of developing severe ARDS during reinfection or late disease progression [6,7], including this population in clinical research is therefore crucial, as their management presents distinct challenges in critical care.

Non-invasive ventilatory strategies such as high-flow nasal cannula (HFNC) and continuous positive airway pressure (CPAP) are commonly used as initial approaches to avoid invasive mechanical ventilation [8,9]. Nonetheless, the available evidence concerning their comparative effectiveness in long COVID ARDS is limited. Previous studies have shown mixed outcomes, with some suggesting that CPAP may reduce the need for intubation and improve short-term oxygenation [8]. In contrast, others report no clear survival benefit compared with HFNC [9,10]. Furthermore, little is known about how pre-intubation ventilatory strategies affect long-term outcomes, including functional sequelae such as impaired pulmonary function, persistent dyspnea, and reduced quality of life among survivors [11,12].

Given the lack of robust data, further investigation is required to clarify optimal ventilatory management in this challenging patient population. The present study sought to evaluate outcomes, sequelae, and treatment strategies in patients with long COVID presenting with severe ARDS, focusing on comparing survival associated with HFNC and CPAP as primary ventilatory support prior endotracheal intubation.

## 2. Materials and Methods

### 2.1. Study Design and Participants

This retrospective cohort study was conducted at the Municipal Clinical Hospital in Timișoara, Romania. It involved patients with long-term COVID-19 who developed severe ARDS and were admitted to the Emergency Department between October 2021 and December 2022.

Inclusion criteria for patients required laboratory-confirmed long COVID-19, characterized by the continuation of symptoms such as fatigue, dyspnea, cognitive impairment, or other sequelae for at least 12 weeks following an acute SARS-CoV-2 infection. These symptoms must not be attributable to alternative diagnoses [5].

**Figure 1:**
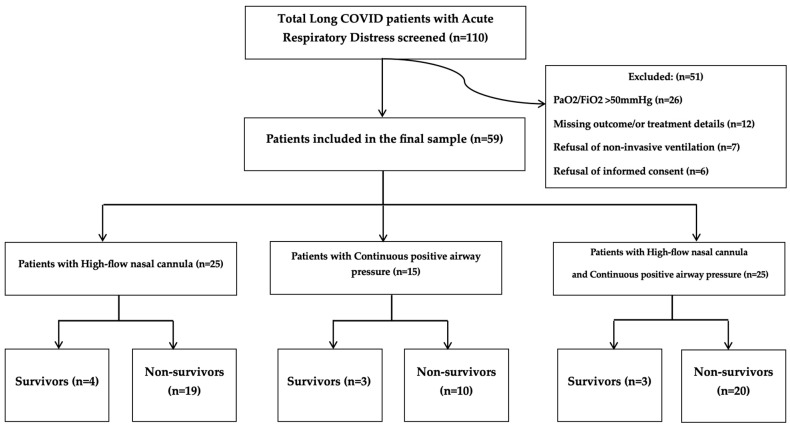
Enrolment and inclusion of potential study participants.

In addition, patients were required to have very severe ARDS, as defined by the Berlin definition, with a PaO_2_/FiO_2_ ratio (Horovitz index) < 50 mmHg [13]. The PaO_2_/FiO_2_ ratio is the ratio of arterial oxygen partial pressure (PaO_2_, in mmHg) to the fraction of inspired oxygen (FiO_2_, expressed as a decimal) and is used to quantify the severity of hypoxemia in ARDS. Severe pulmonary lesions (>80% of lung parenchyma) were confirmed on computed tomography (CT) or chest X-ray at admission. Patients were excluded if they had PaO_2_/FiO_2_ > 50 mmHg (*n* = 26), if treatment or outcome data were missing (*n* = 12), if they refused non-invasive ventilation (*n* = 7) and refusal of informed consent (*n* = 6) Patients included in our cohort had a confirmed diagnosis of long COVID prior to ICU admission and presented with severe ARDS (PaO_2_/FiO_2_ < 50 mmHg) at the time of admission. ARDS was not acquired during hospitalization but represented the reason for admission. After applying these criteria, 59 patients were included and categorized as survivors or non-survivors (Figure 1).

### 2.2. Interventions

Patients were stratified into three groups according to the type of non-invasive ventilation administered: 23 patients received high-flow nasal cannula (HFNC group), 13 patients received continuous positive airway pressure (CPAP group), and 23 patients received combined therapy (HFNC + CPAP group). Patients who required endotracheal intubation during hospitalization were excluded from the analysis.

Standardized pulmonary function testing (spirometry, diffusion capacity), 6-min walk test (6MWT), and validated symptom questionnaires [14] were not systematically performed across the cohort. Therefore, post-ICU sequelae were based primarily on available clinical records and physician assessments rather than on uniform standardized testing.

#### 2.2.1. Continuous Positive Airway Pressure (CPAP)

Patients assigned to CPAP therapy received non-invasive ventilation in CPAP mode per the established national COVID protocols applied in the Intensive Therapy Unit (ICU). Ventilation was delivered using the Dräger Savina 300 ventilator (Dräger, Lübeck, Germany). Eligible patients were required to be awake, adequately sedated, and capable of spontaneous breathing. Before initiation, the procedure was explained in detail, and an appropriately sized interface was selected using the sizing gauge provided with the mask. Interfaces included total face masks (Dimax, sizes M–XL; Sanrai International, Endicott, NY, USA).

Initial ventilator settings consisted of CPAP at 0 cmH_2_O with an FiO_2_ of 1.0. Sedation was achieved using dexmedetomidine. CPAP was then gradually titrated until a reduction in work of breathing was observed. Within approximately 20 min, most patients reached CPAP levels of 10 cmH_2_O with FiO_2_ of 0.6. Thereafter, CPAP was adjusted in increments of 1 cmH_2_O to optimize comfort and patient effort.

#### 2.2.2. High-Flow Nasal Cannula (HFNC)

Patients receiving HFNC therapy were managed according to national COVID-ICU protocols. HFNC was delivered via the Dräger Hi-Flow Star system (Dräger, Germany), which can achieve flow rates of up to 60 L/min.

Treatment was initiated following a standardized protocol: a nasal cannula approximately 50% of the nostril diameter was selected, protective cream was applied to the nostrils, sterile water was connected to the humidifier, and the gas temperature was set at 35 °C with adjustments based on patient comfort. Flow was initiated at the highest level tolerated by the patient, and FiO_2_ was titrated to maintain target oxygen saturation (SpO_2_), with a maximum recommended FiO_2_ of 0.6.

Weaning from HFNC was performed stepwise: after stabilization of oxygenation, FiO_2_ was reduced to a target of 0.30–0.35, while SpO_2_ and respiratory rate were continuously monitored. Flow was subsequently decreased by 10 L/min every 6–8 h until reaching 30 L/min. At this stage, with FiO_2_ maintained at 0.3, patients were transitioned to conventional low-flow oxygen via nasal cannula.

Data on HFNC settings and adjustments were obtained retrospectively from patients’ medical records.

### 2.3. Statistical Analysis

Data analysis was performed using IBM SPSS Statistics version 26.0 (IBM Corp., Armonk, NY, USA). Continuous variables were expressed as mean ± standard deviation (SD) or median and interquartile range (IQR), while categorical variables were summarized as frequencies and percentages. The Shapiro–Wilk test was used to assess the normality of continuous data. For two-group comparisons, the unpaired *t*-test or Mann–Whitney U test was applied for continuous variables, and the χ^2^ test or Fisher’s Exact test for categorical variables. For comparisons across more than two groups, one-way ANOVA or the Kruskal–Wallis test was employed, as appropriate. A *p*-value < 0.05 was considered statistically significant. Kaplan–Meier survival curves with the Log-Rank test were generated to compare outcomes between groups, considering ICU length of stay as the time-to-event variable.

## 3. Results

### 3.1. Patient Demographics

All 59 patients meeting the inclusion criteria were included in the retrospective cohort analysis and were divided into two groups: survivors (n = 10, 15%) and non-survivors (n = 49, 85%). Most patients were between 50 and 80 (78%), with the largest single age group being 60–70 (25%). Among the survivor group, most patients were also aged 50–80 years (63%), although the age distribution differed from that of non-survivors (Table 1). Notably, two patients were younger than 20 years old, one of whom survived and one who did not. There were no patients aged between 20 and 30 years, and all individuals over the age of 80 passed away (Figure 1).

The mortality rate in the ICU was found to be 85% overall, with male patients exhibiting a slightly lower mortality (84%) compared with female patients (88%). Survivors spent a mean of 11.0 ± 10.02 days in the ICU, with a maximum stay of 39 days, whereas non-survivors spent a mean of 6.36 ± 5.85 days in the ICU, with a maximum stay of 33 days. When analyzed by gender, male patients had a mean ICU stay of 7.0 ± 7.5 days, while female patients stayed a mean of 7.2 ± 4.4 days (Table 1).

**Figure 2:**
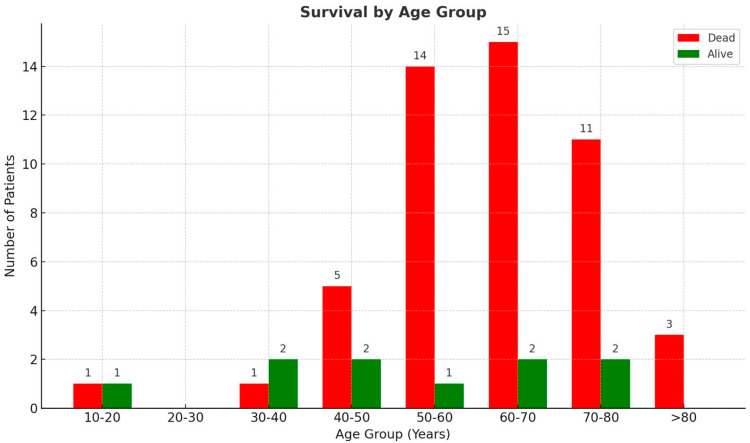
Age of the patients.

Most of the cohort was male, comprising 75% (*n* = 49), while females constituted 25% (*n* = 14). Among the survivors, males represented 80% (*n* = 8) and females 20% (*n* = 2). In contrast, the non-survivor group comprised 74.55% (*n* = 37) males and 25.45% (*n* = 12) females. The youngest male patient was categorized within the 30–40-year-old age range, whereas both individuals in the 10–20-year-old group were female (Figure 2).

### 3.2. Comorbidity Analysis

Patients in the cohort had multiple comorbidities. Survivors had a mean of 3.2 ± 0.6 comorbidities, while non-survivors had a slightly higher mean of 3.33 ± 1.49 (Table 2). Only one patient who died had no comorbidities. The maximum number of comorbidities was six among non-survivors and four among survivors. Mortality did not show a proportional correlation with the number of comorbidities: 100% of patients with fewer than two comorbidities died, 92% of patients with two comorbidities died, 60% of patients with three comorbidities survived, and 90% of patients with more than three comorbidities died.

Among specific comorbidities, diabetes mellitus (DM) was present in 60% of survivors and 56% of non-survivors. Obesity (BMI > 30) was found in 40% of survivors and 42% of non-survivors. Arterial hypertension was more prevalent in survivors (80%) than in non-survivors (71%). Chronic kidney disease (CKD) was observed in 10% of survivors and 16% of non-survivors. Other cardiovascular diseases, including coronary artery disease, heart failure, and chronic venous insufficiency, were more common in non-survivors (58%) than survivors (40%). Pulmonary diseases were rare in both groups (10% of survivors, 11% of non-survivors), while hepatic diseases were present in 30% of survivors and 29% of non-survivors. Oncologic and hematological diseases were found in 30% of survivors and 31% of non-survivors, and neurological disorders affected 20% of survivors and 18% of non-survivors.

When examining the data by gender, it was found that men exhibited a marginally higher mean number of comorbidities (3.33 ± 1.42) compared to females (3.25 ± 1.30), although this variation did not reach statistical significance. Among the ten patients diagnosed with chronic kidney disease, all were male. Pulmonary and hepatic conditions were somewhat more prevalent in men, whereas obesity and diabetes mellitus were more frequently observed in women (56% vs. 37% for obesity; 63% vs. 55% for diabetes). Arterial hypertension was highly prevalent in both genders, though it occurred slightly more often in men (73% vs. 69%). No notable gender differences were identified concerning other cardiovascular diseases, oncological and hematological conditions, or neurological disorders.

### 3.3. Ventilatory Support and Treatment Outcomes

**Figure 3:**
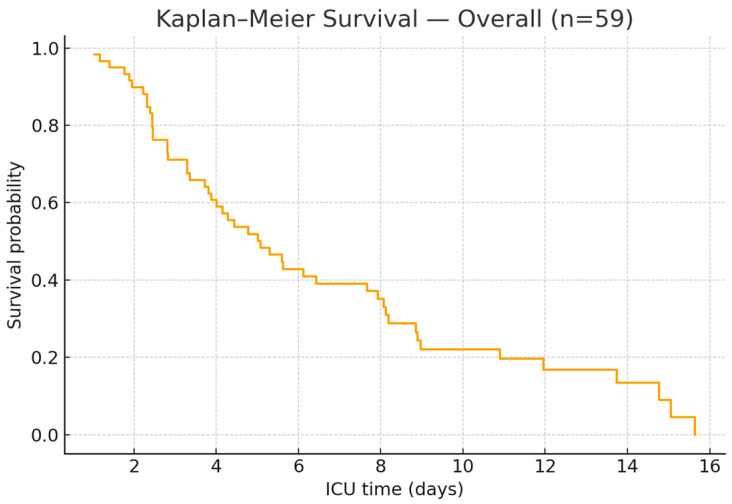
Kaplan–Meier for the overall survival of patients.

Patients were divided into three groups to evaluate mortality using different respiratory support strategies: HFNC-only, CPAP-only, and HFNC + CPAP. In the HFNC-only group (*n* = 23), 17.4% survived and 82.6% died. In the CPAP-only group (*n* = 13), 23.08% survived and 76.92% passed. The HFNC + CPAP group (*n* = 23) had 13% survivors and 87% non-survivors. Gender analysis showed higher mortality for females in the HFNC + CPAP group (100%, *n* = 5). Survival in females was slightly higher in the HFNC-only group (20% vs. 16%) but lower in the CPAP-only group. Male patients had higher survival in the CPAP-only (29% vs. 23.08%) and HFNC + CPAP (17% vs. 13%) groups. Mortality in HFNC-only was similar across genders (17.4% vs. 17%), indicating no significant gender effect. ICU stay varied by treatment: HFNC-only survivors stayed 6.5 ± 3.28 days, CPAP-only 11 ± 2.16 days, and HFNC + CPAP 17 ± 15.9 days. Non-survivors stayed 3.84 ± 2.48 days (HFNC), 5.9 ± 4.16 days (CPAP), and 9.1 ± 7.56 days (HFNC + CPAP). There was no significant difference between CPAP-only survivors and non-CPAP groups (*p* = 0.5). Overall mortality after endotracheal intubation was 86% (*n* = 28). Mortality by prior therapy was 100% in HFNC-only (*n* = 3), 90% in HFNC + CPAP (*n* = 20), and 60% in CPAP-only (*n* = 5), without statistical significance (*p* = 0.124, Figure 3). PaCO_2_ and PaO_2_ at admission did not significantly influence survival. HFNC-only survivors had a mean PaCO_2_ of 35.48 ± 2.25 mmHg, non-survivors 41.87 ± 21.93 mmHg. CPAP-only survivors had 45.55 ± 0.45 mmHg, non-survivors 31.79 ± 4.43 mmHg. HFNC + CPAP survivors had 34.63 ± 6.47 mmHg, non-survivors 33.78 ± 10.05 mmHg (*p* = 0.988). PaO_2_ at admission for HFNC-only survivors was 53.95 ± 24.9 mmHg, CPAP-only survivors 29.85 ± 3.65 mmHg, and HFNC + CPAP survivors 60.23 ± 32.54 mmHg; differences were not statistically significant (*p* = 0.32) (Table 3).

The pooled Kaplan–Meier survival curve of all patients, regardless of ventilatory strategy, shows a steep decline during the first ICU days, reflecting very high early mortality. After this initial period, the curve flattens, suggesting that patients who survived the critical first week or two had a better chance of prolonged ICU survival. The median survival time was very short, around the first week of admission, and the overall ICU survival rate was approximately 15%, in line with the reported 85% mortality in the cohort. A plateau is visible after 20–30 days, indicating that those who remained alive beyond this period were more likely to survive until discharge (Figure 3).

The Kaplan–Meier curves compare three groups of patients: HFNC-only, CPAP-only, and those who received both HFNC and CPAP. At ICU admission, all groups naturally start with a survival probability of 100%. Each step down in the curves represents a death, while flatter segments reflect periods without mortality. The CPAP-only group shows the slowest decline among the three strategies, maintaining the highest survival curve throughout most ICU stays. This suggests a modest short-term survival benefit compared with the other groups.

**Figure 4:**
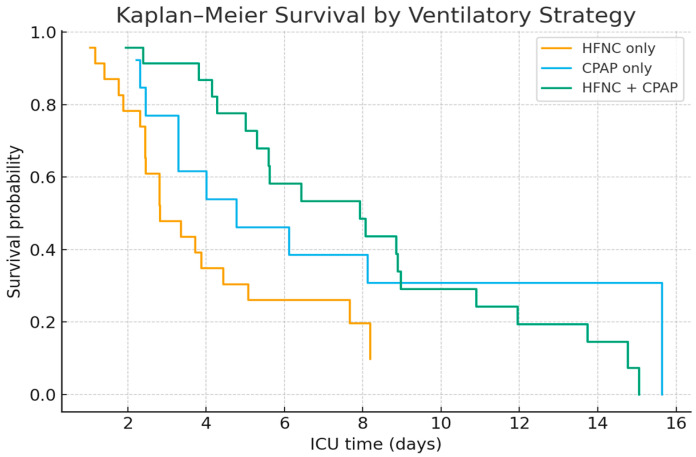
Kaplan–Meier survival by ventilatory strategy and groups.

In contrast, the HFNC-only group demonstrates a steeper drop, indicating higher early mortality. The combined HFNC + CPAP group performs worst, with the sharpest decline, likely reflecting its use in patients with more severe or rapidly worsening conditions. By the end of the ICU follow-up, survival probabilities in all groups converge at low levels. Nevertheless, patients managed with CPAP-only retain a slight survival advantage compared with those treated with HFNC or alternating strategies (Figure 4).

## 4. Discussion

This retrospective study of 59 patients with long COVID and severe ARDS during the second wave revealed similarities and essential differences compared with previously published cohorts. A defining feature of this study is the strict inclusion criterion of a Horovitz quotient (PaO_2_/FiO_2_ ratio) < 50 mmHg, whereas most ARDS cohorts classify severe disease using a threshold of <100 mmHg as per ARDS Definition Task classification [13]. By focusing on patients experiencing extreme hypoxemia, this research offers valuable insights into one of the most critically ill subpopulations. Unsurprisingly, this has resulted in an alarmingly high overall ICU mortality rate of 85%, surpassing the 40–70% mortality typically reported for patients with a PaO_2_/FiO_2_ ratio below 100 mmHg [15,16,17].

The survival curves highlight the extreme early vulnerability of this population, with most deaths occurring within the first week of ICU admission. Patients who survived beyond 20–30 days showed a better chance of recovery, consistent with previous evidence that early deterioration is a critical determinant of outcome [18]. Resource limitations during the second pandemic wave in Romania likely amplified this early mortality. Hospitals were overwhelmed, vaccines were not yet available, and no patients in this study received extracorporeal membrane oxygenation (ECMO), which has been shown to improve outcomes in selected patients with refractory [19]. Moreover, recommendations from Germany and the National Institutes of Health (NIH) support the early intubation of patients presenting with PaO_2_/FiO_2_ levels of ≤100 mmHg [20,21], but such interventions were not consistently feasible under the strained circumstances.

The exceptional 85% ICU mortality observed in this cohort of patients with long COVID and severe ARDS stands in stark contrast to broader population-level data indicating that deaths directly attributable to long COVID remain exceedingly rare. A United States (U.S.) epidemiological study leveraging ICD-10 coding (U09.9) reported that by December 2024, there were 2653 deaths classified as long COVID-19 condition, corresponding to an age-adjusted mortality rate of 0.089 per 100,000 [22]. Similarly, a European analysis during the Omicron wave reported a long COVID condition mortality rate of 1.78 per 100,000, though this rose steeply to 142.74 per 100,000 in individuals aged 75 years and older [23]. These figures suggest that while long COVID can indeed lead to death, such events remain rare at the population level. By contrast, individuals with a history of severe acute infection—particularly those who required hospitalization—demonstrate persistently elevated mortality risk long after recovery. A recent longitudinal study found that survivors of COVID-19 hospitalization exhibited a 29% higher risk of death even three years after infection compared with matched controls [24]. When considered collectively, these results highlight that mortality associated with long COVID is primarily limited to older individuals, those with comorbidities, or patients who have been previously hospitalized. In contrast, the broader population affected by long COVID predominantly experiences morbidity rather than mortality. The notably elevated fatality rate observed in our cohort is likely indicative of the severe nature of respiratory failure (PaO_2_/FiO_2_ < 50 mmHg), categorizing these patients into a distinctly high-risk subgroup within the range of long COVID conditions.

Our study shows that age and comorbidities remained major prognostic factors. Most patients were between 50 and 80 years old, with universal mortality in those over 80. This is consistent with the global consensus that advanced age is among the strongest predictors of poor outcomes in COVID-19 ARDS [25]. Although rare, young patients can also be affected: in this study, two patients between 10 and 20 years were admitted, with one fatal outcome. Chronic comorbidities were ubiquitous, with diabetes, obesity, and hypertension most prevalent, echoing findings from large-scale analyses [26]. Additionally, chronic kidney disease and cardiovascular disease were more commonplace among non-survivors, reaffirming their classification as high-risk conditions [27,28]. Notably, gender disparities in comorbidities were identified: men exhibited hepatic and pulmonary diseases more frequently, whereas women demonstrated elevated rates of obesity and diabetes, reflecting patterns consistent with our national health data from Romania [29].

This divergence underscores an essential distinction between long COVID in the general population—where the primary issue is morbidity—and in patients who are hospitalized or critically ill, where long COVID can present as life-threatening respiratory failure. Long-term research further reveals that individuals who were previously hospitalized due to COVID-19 experience an increased risk of mortality for several years following infection, with an excess mortality risk of 29% even three years after hospitalization [24]. Collectively, these insights imply that while the majority of those suffering from long COVID endure ongoing disabilities rather than fatal outcomes, mortality emerges as a critical concern for older patients with comorbidities and severe respiratory complications, similar to those in our study group.

In the cohort examined in this study, a strong association was observed between ventilatory strategy and patient outcomes. Patients receiving CPAP-only demonstrated improved survival rates (23%) compared to those treated with HFNC-only (17%) or alternating HFNC + CPAP (13%), although the differences did not achieve statistical significance due to the limited sample size. This observation is consistent with international guidelines that advocate for the use of CPAP over HFNC in cases of severe ARDS, while both modalities are preferred over traditional oxygen therapy [30]. Mortality following intubation was exceptionally high (86%), slightly exceeding overall cohort mortality, consistent with other reports that invasive ventilation carries a poor prognosis in severely ill COVID-19 patients [15]. Notably, survivors in the CPAP-only group had the lowest mean PaO_2_ at admission, suggesting that factors beyond initial oxygenation—such as timing, therapy tolerance, and patient selection—likely influenced outcomes [31]. When comparing these findings to long COVID mortality rates in the general population, they reveal a stark contrast in risk profiles. Collectively, these data emphasize that while the morbidity associated with long COVID is prevalent, its lethality remains infrequent within the broader population. Conversely, our cohort constitutes a meticulously chosen subset—patients experiencing severe ARDS and profound hypoxemia (PaO_2_/FiO_2_ < 50 mmHg)—for whom mortality rates are significantly heightened, irrespective of the type of ventilation employed. This underscores that fatalities associated with long COVID are predominantly limited to critically ill subgroups, with CPAP providing merely a slight advantage in survival amidst an environment characterized by predominantly unfavorable outcomes.

This study highlights the complex interplay between disease severity, comorbid conditions, and constrained resources throughout the pandemic. The significant mortality rate highlights the clinical difficulties associated with managing patients exhibiting PaO_2_/FiO_2_ levels below 50 mmHg, a subgroup that has been infrequently addressed in earlier studies. While CPAP seemed to provide a slight survival benefit, the overall outcomes were predominantly influenced by factors such as severe hypoxemia, age, and the burden of comorbidities. These findings underscore the importance of promptly identifying high-risk patients, appropriately escalating respiratory support, and ensuring access to advanced treatments like ECMO when clinically necessary.

This study highlights the interaction among disease severity, comorbid conditions, and constrained resources throughout the pandemic. The significant mortality rate highlights the clinical difficulties associated with managing patients exhibiting PaO_2_/FiO_2_ levels below 50 mmHg, a subgroup that has been infrequently addressed in earlier studies. Although CPAP seemed to provide a slight survival benefit, the overall outcomes were predominantly influenced by factors such as severe hypoxemia, age, and the burden of comorbidities. These findings underscore the importance of promptly identifying high-risk patients, appropriately escalating respiratory support, and ensuring access to advanced treatments like ECMO when necessary [32] demonstrated significant benefits in reducing fatigue, improving pulmonary function, and enhancing quality of life in patients with post-COVID condition. These findings support the integration of multidisciplinary rehabilitation programs into the continuum of care for long COVID patients recovering from severe illness.

Additional insights can be drawn from research on other chronic respiratory diseases. For example, Florian et al. [33], showed that targeted respiratory muscle training not only improved respiratory outcomes but also enhanced functional performance, including balance and gait. Although these findings come from a COPD population, the underlying mechanisms may be relevant to post-COVID rehabilitation, suggesting that respiratory muscle training could further support recovery of physical function and quality of life in this population.

Taken together, our findings and the growing body of evidence emphasize the need for a comprehensive management strategy that combines early recognition and escalation of care in the ICU with structured, multidisciplinary rehabilitation interventions during recovery. Such an approach may offer the best opportunity to reduce mortality, mitigate long-term sequelae, and improve the quality of life of patients with long COVID complicated by severe ARDS.

### Study Strengths, Limitations and Future Research

Several strengths of this study should be acknowledged. All patients meeting the inclusion criteria were captured, minimizing selection bias and ensuring a complete cohort. The study provides a detailed description of demographics, comorbidities, and outcomes stratified by ventilatory strategy, and the use of Kaplan–Meier survival curves allowed a temporal understanding of mortality trends. The integration of gender—and age-stratified analyses added further depth, permitting exploration of population subgroups.

However, limitations must also be considered. The relatively small sample size, particularly of survivors, limited statistical power and the generalizability of subgroup comparisons. As a single-center retrospective study, findings are subject to documentation bias, unmeasured confounding, and may not reflect practices in other healthcare systems. Resource constraints during the second COVID-19 wave in Romania—such as the absence of ECMO and limited capacity for timely intubation—likely influenced outcomes. Finally, the observational design precludes conclusions about causality between ventilatory strategies and survival, as patients receiving dual modalities were likely more severely ill at baseline.

Future research should focus on overcoming the limitations of this study by employing larger, multicenter prospective cohorts that implement standardized treatment protocols. Incorporating validated metrics for severity of illness, such as the Sequential Organ Failure Assessment (SOFA) score—which evaluates the extent of dysfunction across major organ systems [34], and the Acute Physiology and Chronic Health Evaluation II (APACHE II) score, which combines acute physiological disturbances with chronic health conditions to estimate mortality risk [35], would significantly enhance risk stratification and improve prognostic modeling. Well-structured randomized controlled trials are essential to establish causality and refine international guidelines, comparing CPAP, HFNC, and combined approaches.

Furthermore, long-term follow-up studies that assess not only survival rates but also quality of life, functional recovery, and post-ICU morbidity would yield a more comprehensive understanding of survivorship among patients suffering from long COVID–associated ARDS.

Follow-up assessments relied on clinical documentation rather than standardized functional tests or questionnaires, which limits precision in characterizing sequelae. In addition, post-discharge mortality and rehospitalization were not systematically captured. Future studies should include structured long-term follow-up with validated tools and comprehensive outcome reporting.

## 5. Conclusions

In this study of patients with long COVID-related severe ARDS (PaO_2_/FiO_2_ < 50 mmHg), survival outcomes differed numerically between CPAP, HFNC, and combined therapies, but these differences did not achieve statistical significance. Although CPAP showed a trend toward improved survival, the data do not establish superiority and should be regarded as hypothesis-generating.

These results highlight the need for caution in interpreting non-invasive support strategies in this high-risk subgroup and reinforce the importance of larger, multicenter investigations with broader inclusion criteria to validate and extend these preliminary findings.

## Figures and Tables

**Table 1:** General demographic parameters.

Parameter	Survivor (*n* = 10, 15%)	Non-Survivor (*n* = 49, 85%)
Female (n, %)	2 (20%)	12 (25.45%)
Male (n, %)	8 (80%)	37 (74.55%)
Days in ICU (mean ± SD, max)	11 ± 10.02 (max 39)	6.36 ± 5.85 (max 33)
Smoking (n, %)	0	2 (4%)

**Table 2:** Patient comorbidities.

Comorbidities	Survivor (*n* = 10)	Non-Survivor (*n* = 49)
Number of comorbidities (mean ± SD)	3.2 ± 0.6	3.33 ± 1.49
Range of comorbidities (min–max)	2–4	0–6
Diabetes Mellitus (n, %)	6 (60%)	31 (56%)
BMI > 30 (n, %)	4 (40%)	23 (42%)
Arterial hypertension (n, %)	8 (80%)	32 (71%)
Pulmonary diseases (n, %)	1 (10%)	6 (11%)
Other cardiovascular diseases (n, %)	4 (40%)	32 (58%)
Hepatic diseases (n, %)	3 (30%)	16 (29%)
Onco- and hematological disease (n, %)	3 (30%)	17 (31%)
Neurological disorders (n, %)	2 (20%)	10 (18%)
Chronic kidney disease (n, %)	1 (10%)	9 (16%)

**Table 3:** Patients’ therapy.

Patients’ Therapy		
**Therapy (HFNC)**	**Survivor**	**Non-Survivor**
Number of patients (*n*, %)	4 (17.4%)	19 (82.6%)
On ICU (in days) (mean, SD)	6.5 ± 3.28	3.84 ± 2.48
Endotracheal intubation (*n*, %)	0	3 (15.79%)
P_a_CO_2_ at admission (mean, SD)	35.48 ± 2.25 mmHg	41.87 ± 21.93 mmHg
P_a_O_2_ at admission (mean SD)	53.95 ± 24.9 mmHg	55.35 ± 26.49 mmHg
**Therapy (CPAP)**		
Number of patients (*n*, %)	3 (23.08%)	10 (76.92%)
On ICU (in days) (mean, SD)	11 ± 2.16	5.9 ± 4.16
Endotracheal intubation (*n*, %)	2 (66.67%)	3 (30%)
P_a_CO_2_ at admission (mean, SD)	45.55 ± 0.45 mmHg	31.79 ± 4.43 mmHg
P_a_O_2_ at admission (mean SD)	29.85 ± 3.65 mmHg	54.83 ± 25.25 mmHg
**Therapy (HFNC + CPAP)**		
Number of patients (*n*, %)	3 (13.04%)	20 (86.96%)
On ICU (in days) (mean, SD)	17 ± 15.9	9.1 ± 7.56
Endotracheal intubation (*n*, %)	2 (66.67%)	18 (90%)
P_a_CO_2_ at admission (mean, SD)	34.63 ± 6.47 mmHg	33.78 ± 10.05 mmHg
P_a_O_2_ at admission (mean SD)	60.23 ± 32.54 mmHg	48.12 ± 30.63 mmHg

## Data Availability

The datasets are not publicly available; however, Florina Buleu or Daian Popa may provide de-identified data upon request.
